# Reliability and validity of the Korean version of MAPPIN’SDM for patients and healthcare providers

**DOI:** 10.1371/journal.pone.0347018

**Published:** 2026-09-03

**Authors:** Heeseung Choi, Jongeun Lee, Sang-Ho Yoo, Junggeun Ahn, Youngeun Park, Hannah Kim, Jürgen Kasper

**Affiliations:** 1 College of Nursing, Seoul National University, Seoul, Republic of Korea; 2 Research Institute of Nursing Science, Seoul National University, Seoul, Republic of Korea; 3 College of Nursing, Chungbuk National University, Cheongju, Republic of Korea; 4 Research Institute of Nursing Science, Chungbuk National University, Cheongju, Republic of Korea; 5 Department of Medical Humanities and Ethics, College of Medicine, Hanyang University, Seoul, Republic of Korea; 6 College of Nursing, Jeju National University, Jeju, Republic of Korea; 7 Health and Nursing Research Institute, Jeju National University, Jeju, Republic of Korea; 8 Center for World-leading Human-Caring Nurse Leaders for the Future by Brain Korea 21 (BK21) Four Project, Seoul National University, Seoul, Republic of Korea; 9 Department of Nursing and Health Promotion, Faculty of Health Sciences, Oslo Metropolitan University, Oslo, Norway; University of Pennsylvania, UNITED STATES OF AMERICA

## Abstract

**Background:**

Shared decision making (SDM) integrates the expertise of healthcare providers with the values and preferences of patients. The Multifocal Approach to Sharing in Shared Decision-Making (MAPPIN’SDM) based on a six-step SDM model, has shown robust reliability and validity in diverse settings. This study aimed to validate the Korean version (K-MAPPIN’SDM) and assess its applicability in Korean clinical practice.

**Methods:**

The K-MAPPIN’SDM was developed through a translation and cultural adaptation process including forward translation, synthesis, blind back translation, semantic equivalence review, and multidisciplinary evaluation for accuracy in the Korean context. The instrument was piloted with six physicians and 288 patients involved in SDM. For construct validity, exploratory factor analysis (EFA) and confirmatory factor analysis (CFA) were conducted. Psychometric evaluation included Pearson’s correlation, Guttman split-half coefficient, and Cronbach’s alpha. Criterion validity was assessed using the 9-Item Shared Decision-Making Questionnaire (SDM-Q-9), SDM-Q-Doc, and Communication Assessment Tool (CAT).

**Results:**

The final K-MAPPIN’SDM was successfully developed through a scientifically rigorous and culturally sensitive translation and validation process. EFA and CFA supported a one-factor model for both versions of K-MAPPIN’SDM, though model fit was better for the patient version. Internal consistency of the K-MAPPIN’SDM was high (Cronbach’s α = 0.94 for healthcare provider version; 0.87 for patient version). Guttman coefficients were 0.90 and 0.73, respectively. The provider version correlated strongly with SDM-Q-Doc (r = 0.88, p < .001); the patient version correlated with SDM-Q-9 (r = 0.58, p < 0.001) and CAT (r = 0.47, p < .001).

**Conclusions:**

The K-MAPPIN’SDM is a psychometrically sound tool for assessing SDM. This scale has the potential to be widely used to assess SDM in Korea. K-MAPPIN’SDM can serve as a tool to assess current state of SDM implementation from diverse perspectives in Korea and provide framework for training healthcare providers in SDM practices.

## 1. Introduction

Shared decision-making (SDM) is an interactive process in which patients and healthcare professionals share evidence-based clinical information to make the best medical decisions reflecting patients’ preferences [1]. By enhancing patients’ understanding of their illness and treatment processes, SDM enables active participation in their care. Decisions aligning with patient values improve treatment adherence and health outcomes [2]. Models developed to implement SDM in clinical practice include the SHARE Approach [3], the Three-Talk Model [4], and the Six-Step SDM Model [5].

With the development of SDM, the necessity for assessment tools to evaluate it has emerged. Assessment tools have been developed to ensure proper implementation and evaluation of SDM [2,6]. Evaluating SDM is essential to measure its effectiveness in clinical practice and understand its impact on patient health outcomes [7,8]. A comprehensive assessment of SDM includes examining the decision-making process quality and its outcomes to ensure that the SDM is implemented as intended [9]. Moreover, reliable and valid SDM assessment tools are crucial in supporting healthcare improvements, guiding clinical practice, and facilitating policy formulation and implementation [2].

Recognizing that the perceptions, expectations, and evaluations of SDM may differ among patients, healthcare providers, and other key stakeholders, assessment tools have been developed to capture multiple group perspectives. Specifically, these tools are designed for patients, their families, and healthcare providers, ensuring a more comprehensive evaluation of SDM. Moreover, tools have been developed to assess various aspects and stages of SDM, such as its antecedents, processes, qualities, and outcomes [6]. Representative tools for evaluating the process and outcomes of SDM include the 9-Item Shared Decision-Making Questionnaire (SDM-Q- 9) [10], Observing Patient Involvement in Shared Decision-Making (OPTION) [11], and Multifocal Approach to Sharing in Shared Decision-Making (MAPPIN’SDM) [12].

The SDM assessment tools used in South Korea include a tool developed by the researcher [13], which compiled items from various instruments used in previous studies, the Korean Shared Medical Decision-Making Scale [14], the Korean-translated version of SDM-Q-9 [10,15], and the Korean-translated version of the Modified Version of the Perceived Involvement in Care Scale (M-PICS) [16,17]. Among these, the SDM-Q-9 adapted by Park et al. [15], and the scale developed by Jo [14] are the most widely used.

The “Korean Shared Medical Decision-Making Scale,” developed by Jo [14], consists of 34 items that assess attitudes toward SDM from the perspectives of patients, families, and healthcare providers. This tool reflects Korea’s traditional culture, which emphasizes family interests and collective decision-making, and includes seven subdomains: information sharing, support system establishment, duty of explanation, autonomy, timing, family involvement, and respect for dignity. However, because the tool was developed specifically for end-of-life setting, its applicability to broader healthcare settings and a variety of health issues is limited. Moreover, since the tool reflects a Korean healthcare culture that traditionally places greater emphasis on family involvement rather than individual patient preferences, there remains a need for an SDM assessment tool that prioritizes patient-centered decision-making and autonomy.

The SDM-Q-9 also has notable limitations. Unlike the MAPPIN’SDM, it does not systematically assess each step of the SDM process, such as defining the problem, explaining the benefits and harms of each option, exploring patient preferences and needs, or indicating the decision. In addition, no validation studies of the Korean version of the SDM-Q-9 have been published to date.

In conclusion, among the SDM assessment tools currently used in South Korea, there is a lack of tools that can be widely applied to various health conditions and have undergone systematic development and validation. Moreover, no comprehensive Korean SDM assessment tool incorporates subjective evaluations by key stakeholders (e.g., patients and healthcare providers) and objective evaluations by external observers.

MAPPIN’SDM is a comprehensive and structured evaluation tool that measures the extent of SDM through a multifocal approach, integrating the self-perception of both patients and healthcare providers, along with an objective evaluation by an observer [12]. This measure consists of three instruments tailored to patients, healthcare providers, and observers. Unlike conventional SDM assessment tools relying on patient or provider self-reporting, the MAPPIN’SDM incorporates multiple perspectives, allowing for a more objective and holistic evaluation of the SDM process. Designed to assess each step of the SDM process [5], it enables a deeper analysis of how decision-making unfolds between patients and healthcare providers, ensuring a triangulated, evidence-based assessment of SDM quality. Originally developed in German by Kasper et al. (2012), the MAPPIN’SDM was translated into English and validated in Norway with an improved observer version [18]. Its application across numerous clinical contexts, including oncology, chronic disease management (e.g., multiple sclerosis), and dentistry, demonstrates its versatility and robustness as an SDM assessment instrument [9].

As an initial phase of this research, this study focused on the translation and validation of the patient and healthcare provider versions of the instrument. By evaluating the psychometric properties of these two instruments, this study seeks to establish a reliable and valid tool for measuring SDM in Korea, ultimately contributing to the development of a comprehensive SDM model and assessment framework tailored to the Korean healthcare environment. This study aimed to develop and validate the Korean version of the MAPPIN’SDM (K-MAPPIN’SDM) to assess SDM in Korean clinical settings.

## 2. Methods

### 2.1. Design

This cross-sectional study comprises two phases: a scientifically rigorous and culturally sensitive translation of the MAPPIN’SDM into Korean, followed by validation of the instrument in a Korean clinical setting.

### 2.2. Participants

Participants in the study were physicians and adult patients (aged ≥18 years) recruited through convenience sampling from a university hospital, all of whom participated in outpatient consultations involving treatment-related decision-making. During the recruitment period, physicians from the departments of endocrinology, orthopedics, and nephrology agreed to participate in the study, and patients receiving care in these departments were subsequently recruited. To confirm decision-making, both physicians and patients briefly documented the decisions made during the consultation in their respective questionnaires.

The original developer of the measure does not specify a minimum sample size for validating the healthcare provider version as long as the variability of SDM interactions is included. For the patient version, the sample size was determined based on Comrey and Lee [19], which suggested that 200 cases were fair and 300 were good for validation. In total, 288 outpatient consultations were analyzed. For the healthcare provider version, the unit of analysis was the SDM encounter rather than the individual physician. Although six physicians participated, each physician completed the healthcare provider version of the MAPPIN’SDM across multiple outpatient consultations, resulting in 288 healthcare provider–rated SDM encounters.

### 2.3. Measures

#### 2.3.1. Demographic data.

For physicians, data were collected on age, sex, department, clinical experience, and training in SDM and communication. For the patients, information included age, sex, marital status, household composition, occupation, education, socioeconomic status, and decision-making preferences. Decision-making preferences were measured using the Korean version of the Control Preference Scale (CPS), which assesses the extent to which patients prefer to make treatment decisions or delegate them to their physicians [20].

#### 2.3.2. MAPPIN’SDM.

The structure of MAPPIN’SDM is explained based on three perspectives (i.e., patients, healthcare providers, and observers), two constructs (i.e., behaviors and results), and three units (i.e., patients, healthcare providers, and the healthcare provider–patient dyad).

The initial version of the MAPPIN’SDM was developed to assess both behavior and result constructs in patients and healthcare providers. In the present study, however, a revised version recommended by the original developer was used, which exclusively measured the result construct. This modification was made to minimize response bias that may arise when patients and healthcare providers assess their own behaviors. It highlights the appropriateness of using an observer questionnaire for a more objective assessment of behavior constructs. In other words, even when patients and healthcare providers evaluate only the result construct of SDM, it is still possible to indirectly infer that certain behaviors have been performed. Furthermore, this approach is intended to offer a more reliable assessment than direct behavior measurement. This revision is specifically significant because it is supposed to reduce subjective bias in the SDM process and enhances the objectivity of the evaluation.

In this study, a K-MAPPIN’SDM for patients and healthcare providers was developed and evaluated for its psychometric properties. Each questionnaire comprised 11 indicators assessed using a 5-point Likert scale ranging from “strongly disagree” (0) to “strongly agree” (4). Higher scores indicate greater levels of SDM. These 11 indicators systematically evaluate the six steps of the SDM process. The first step, “defining the goal of consultation,” was assessed using Indicator 1, “defining the problem.” The second step, “explaining the need for patient participation,” was evaluated using Indicator 2, “key message.” The third step, “explaining the benefits and harms of each option,” was assessed using Indicators 3–5, which measure the quality of options regarding structure, content, and information quality. The fourth step, “exploring patient preferences and needs,” was evaluated using Indicator 6, “expectations and worries.” The fifth step, “making a shared decision,” was assessed using Indicator 7, “indicating the decision.” The sixth step, “implementing the decision,” was evaluated using Indicator 8, “follow-up arrangements.” Finally, Indicators 9–11 served as additional criteria in the decision-making process, assessing the overall communication and mutual understanding between patients and healthcare providers.

#### 2.3.3. SDM-Q-9/SDM-Q-Doc.

The instruments used to evaluate the criterion validity of the MAPPIN’SDM were the SDM-Q-9 physician and patient version. The SDM-Q-9 (patient version) and SDM-Q-Doc (physician version) assess SDM from both patients’ and physicians’ perspectives [10]. Both consist of nine items rated on a 6-point Likert scale (0 = “completely disagree” and 5 = “completely agree”), with total scores ranging from 0 to 45, where higher scores indicate a greater level of SDM. This study used the Korean versions of the SDM-Q-9 (patient version) and SDM-Q-Doc (physician version), and the Cronbach’s alphas for these measures were 0.95 and 0.87, respectively [21]. The original versions had Cronbach’s alphas of 0.94 (SDM-Q-9) and 0.88 (SDM-Q-Doc) [10,22].

#### 2.3.4. Communication Assessment Tool.

Another tool employed in this study to evaluate the criterion-related validity of the MAPPIN’SDM scale was the Communication Assessment Tool (CAT). The CAT was developed to measure patients’ perceptions of physicians’ interpersonal and communication skills during outpatient consultations [23]. It is designed to be completed immediately after consultation and consists of 15 items rated on a 5-point Likert scale (1 = “strongly disagree” and 5 = “strongly agree”). We used the Korean version of the CAT, which has a Cronbach’s alpha of 0.96 [24]. The original version had a Cronbach’s alpha of 0.96 [23].

### 2.4. Procedure

#### 2.4.1. Translation and linguistic validation.

The translation process was conducted with the approval of the original developer, Dr. Kasper, and was systematically refined through multiple consultations via virtual meetings and email correspondence. The translation and validation procedures followed the guidelines proposed by Sousa and Rojjanasrirat [25]. Two professional translators independently translated the original version into Korean, one with expertise in the field of medicine. A panel of five researchers reviewed both translations to resolve inconsistencies and refined expressions to produce a preliminary Korean version. Two independent professional translators, with no prior exposure to the preliminary version, back-translated it into English to ensure accuracy. The independence between translators and back-translators was strictly maintained. The two back-translations were then compared with the original, and the original developer confirmed the absence of semantic distortions. Based on this feedback, minor modifications were made to the wording and terminology. A final expert review was conducted by four specialists in ethics, linguistics, medical humanities, and public policy. After incorporating expert feedback and discussions among the research team, the final version of the K-MAPPIN’SDM was established.

The original developer’s feedback and expert consultations led to several modifications. For example, in Item 2 of the patient version, the translation was revised to clarify that the patient, rather than the healthcare provider, is the decision-maker when considering medical options. In Item 3c, the phrase “benefits and harms” was adjusted to prevent its meaning from being limited to “advantages and disadvantages.” Furthermore, regarding the translation of “option,” the first occurrence of the term was modified to include both the transliterated term and its Korean equivalent in parentheses to ensure that patients clearly understand that “option” means all possible choices in the decision-making process.

### 2.5. Data collection

Data were collected from July 31 to August 26, 2024, at a university hospital in Seoul, South Korea. Participation was voluntary, and only physicians from departments that agreed to participate, along with patients receiving care in those departments, were included in the study. Written informed consent was obtained from the participating physicians, and their cooperation was secured for data collection.

The research team remained on site during outpatient consultation hours, provided patients with a detailed explanation of the study, and obtained written informed consent prior to data collection. Because this study aimed to evaluate SDM involving both patients and physicians, data were collected from both groups. After a patient completed the questionnaire, the research team provided the healthcare provider questionnaire to the respective physician. Upon completion of data collection, participants received a mobile gift card as compensation.

### 2.6 Data analysis

The collected data were analyzed using IBM SPSS Statistics version 29.0 and R (version 4.4.1). Before the main analyses, the dataset was screened for missing values. No missing values were identified in the variables included in the psychometric analyses; therefore, no case deletion due to missingness or imputation procedure was required.

The participants’ demographic data were examined using frequencies, percentages, means, and standard deviations. To assess the internal consistency of the measurement, Guttman’s split-half reliability coefficients and Cronbach’s alpha coefficients were calculated. Moreover, item analysis was conducted by examining the corrected item-total correlation coefficients. Although items with low correlations are commonly considered for removal during scale development [26], each item in this scale is designed as an essential component of the SDM process, with equal weighting across all items [27]. Therefore, item deletion was not applied in this study. Descriptive statistics, including the mean and standard deviation for each item, were calculated.

For construct validity, exploratory factor analysis (EFA) and confirmatory factor analysis (CFA) were conducted. Exploratory and confirmatory analyses were conducted using the same dataset. The exploratory analysis was intended to examine the structural coherence of the predefined indicators rather than to inductively derive latent dimensions, and the confirmatory analysis was performed as a supplementary evaluation of model fit based on the theoretically established structure of the original instrument.

EFA was conducted to examine the structural coherence of the predefined indicators. The Kaiser–Meyer–Olkin (KMO) measure and Bartlett’s test of sphericity were used solely to assess the suitability of the data for exploratory analysis. To examine the structural coherence among the predefined indicators of the SDM process, principal component analysis (PCA) was conducted. The MAPPIN’SDM (clinician and patient versions) is conceptualized as a formative measurement instrument in which each item represents a distinct and theoretically essential SDM process indicator. Because the indicators are non-interchangeable and retained by design, no item was considered for deletion and dimensional reduction procedures were not applied. For clearer interpretation and separation of the component structure, an orthogonal Varimax rotation was employed. Loadings of ≥ 0.40 were considered salient for interpretative purposes. Communalities and cross-loadings were examined to evaluate the contribution of each indicator to the component structure.

Because the number of indicators was fixed a priori and not subject to empirical reduction, eigenvalues and scree plots were inspected descriptively to understand the component structure, but they were not used as the sole basis for item retention, item deletion, or dimensional reduction. Any emergence of multiple components was interpreted as clustering among distinct SDM process elements rather than evidence of separable latent constructs. Any emergence of multiple components was interpreted as clustering among distinct SDM process elements rather than evidence of separable latent constructs.

Subsequently, CFA was performed to assess the model fit, which was evaluated based on the following fit indices: Tucker-Lewis index (TLI ≥ 0.90), comparative fit index (CFI ≥ 0.90), root mean square error of approximation (RMSEA ≤ 0.08), and standardized root mean square residual (SRMR ≤ 0.08) [28,29]. To examine criterion-related validity, Pearson’s correlation coefficient was applied to examine the relationship between the healthcare provider version of the K-MAPPIN’SDM and SDM-Q-Doc. Similarly, criterion-related validity of the patient version was examined by assessing its correlation with both the SDM-Q-9 and CAT.

### 2.7. Ethical considerations

This study was approved by the Institutional Review Board (IRB) of S. University Hospital (IRB No. 2407-115-1554). Patients were recruited in consultation with physicians in endocrinology, orthopedics, and nephrology, and those who met the eligibility criteria were selected. Participants received a detailed explanation of the study’s purpose, procedures, anonymity, and data protection measures along with a study explanation document. Only those who provided written voluntary consent were enrolled. The participants were informed that they could withdraw at any time without consequences, that the study was independent of their clinical care, and that refusal to participate would not affect their treatment. The physicians who participated in the study confirmed that they had no conflicts of interest with the research team.

### 2.8. Inclusivity in global research

This study ensured the engagement of the communities and that the communities were valued throughout the research process. Additional information regarding the ethical, cultural, and scientific considerations specific to inclusivity in global research is included in the Supporting Information (S1 Checklist).

## 3. Results

### 3.1. Characteristics of study participants

#### 3.1.1. Healthcare providers.

Six healthcare providers participated in this study, including two endocrinologists (33.3%), three orthopedic surgeons (50.0%), and one nephrologist (16.67%). The average clinical experience was 16.50 ± 9.38 years. Only one participant (16.67%) had received structured training in communication or SDM (Table 1).

**Table 1:** General characteristics of healthcare providers (n = 6).

Characteristics	Category	n (%) or Mean±SD
Sex	Male	5 (83.3)
	Female	1 (16.7)
Department	Endocrinology	2 (33.3)
	Orthopedics	3 (50.0)
	Nephrology	1 (16.7)
Total years of clinical experience		16.50 ± 9.38
Completion of communication training	Yes	1 (16.7)
	No	5 (83.3)
Completion of SDM training	Yes	1 (16.7)
	No	5 (83.3)

Note: SDM = Shared decision-making

#### 3.1.2. Patients.

A total of 288 patients (94 men [32.6%] and 194 women [67.4%]) participated in the study (the mean age was 51.91 ± 14.86 years). The age distribution of patients was as follows: 18–29 years (n = 28), 30–39 years (n = 33), 40–49 years (n = 61), 50–59 years (n = 61), 60–69 years (n = 76), and 70 years or older (n = 29). The distribution of patients by department involved in the SDM process was as follows: orthopedic surgery, 110 patients (38.2%); endocrinology, 95 patients (33.0%); and nephrology, 83 patients (28.8%). Regarding treatment decision-making preferences, the most preferred option was to make decisions with the physician, chosen by 134 patients (46.5%). This was followed by patients who preferred to carefully consider their opinions but let the physician make the final decision (87 patients, 30.2%) (Table 2).

**Table 2:** General characteristics of patients (n = 288).

Characteristics	Category	n (%) or Mean±SD
Sex	Male	94 (32.6)
	Female	194 (67.4)
Age		51.91 ± 14.86
Department	Endocrinology	95 (33.0)
	Orthopedics	110 (38.2)
	Nephrology	83 (28.8)
Marital status	Single	68 (23.6)
	Married	195 (67.7)
	Separated	0 (0.0)
	Divorced	8 (2.8)
	Widowed	17 (5.9)
Current occupation	Unemployed	43 (14.9)
	Full-time employee	94 (31.6)
	Contract employee	17 (5.9)
	Self-employed	47 (16.3)
	Temporary/Part-time worker	2 (0.7)
	Student	10 (3.5)
	Homemaker	61 (21.2)
	Other	17 (5.9)
Residence type	Living alone	50 (17.4)
	Living with a spouse or children in your own home	191 (66.3)
	Living in a parent’s or child’s home	41 (14.2)
	Living with relatives or friends	1 (0.3)
	Other	5 (1.7)
Education level	Middle school graduate or below	33 (11.5)
	High school graduate	78 (27.1)
	Bachelor’s degree	143 (49.7)
	Master’s or doctoral degree	34 (11.8)
Socioeconomic status	Upper	8 (2.8)
	Upper-middle	71 (24.7)
	Middle	162 (56.3)
	Lower-middle	35 (12.2)
	Lower	12 (4.2)
Control Preference Scale	I prefer to make the final selection about which treatment I will receive.	4 (1.4)
	I prefer to make the final selection of my treatment after seriously considering my doctor’s opinion.	45 (15.6)
	I prefer that my doctor and I share responsibility for deciding which treatment is best for me.	134 (46.5)
	I prefer that my doctor makes the final decision about which treatment will be used, but seriously considers my opinion.	87 (30.2)
	I prefer to leave all decisions regarding my treatment to my doctor.	18 (6.3)

### 3.2. Descriptive statistics by items

The analysis of the 11 items on the healthcare provider version of the K-MAPPIN’SDM indicated that the item-specific mean scores ranged from 3.17 to 3.49, with standard deviations between 0.58 and 0.80. The three items with the highest mean scores were Item 11: “I understood the patient’s viewpoint,” Item 10: “The patient understood the information that I gave him,” and Item 6: “The patient’s personal expectations and fears went into the decision.” Conversely, the three items with the lowest mean scores were Item 4: “The patient now knows the pros and cons of the different decision options,” Item 3: “The discussion of the options was structured in a way that is easy to understand and easy to remember,” and Item 5: “Based on the information the patient developed realistic expectations on probabilities of benefit and harms of each option.”

**Fig 1:**
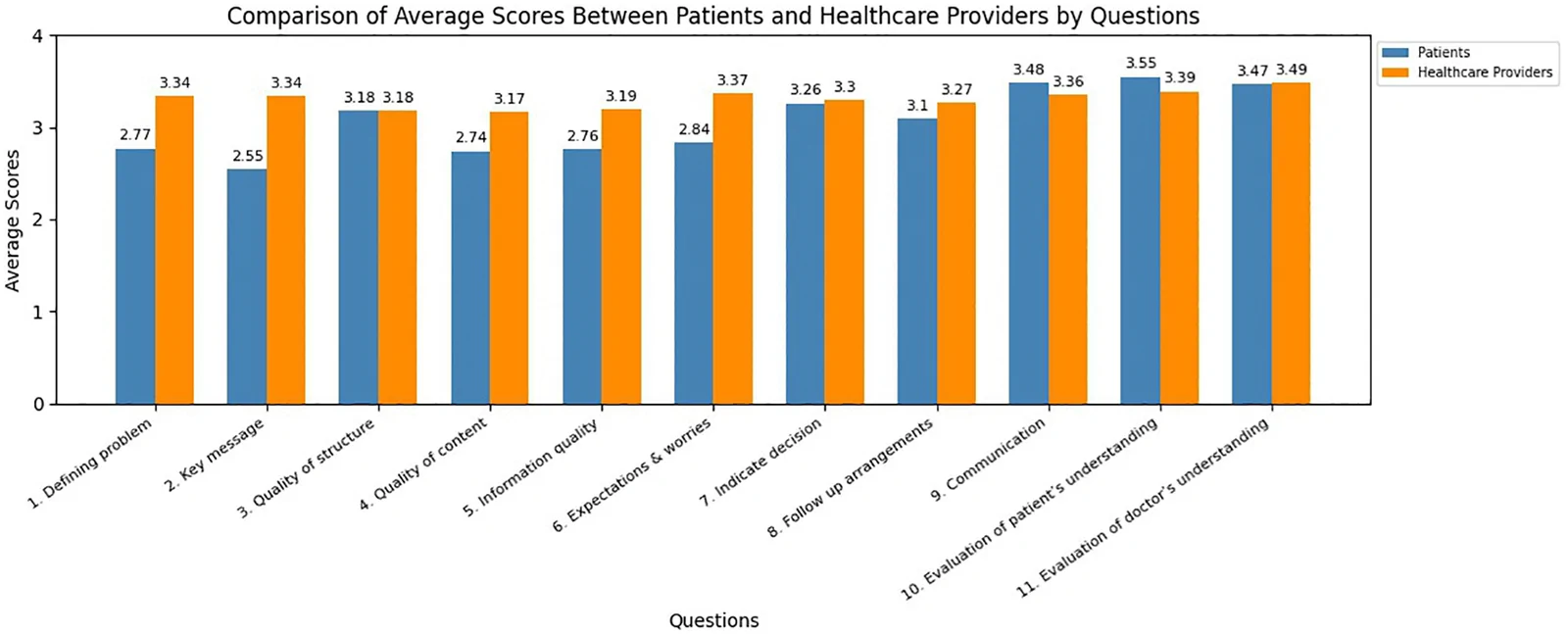
Comparison of average MAPPIN’SDM item scores between healthcare providers and patients.

The analysis of the 11 items on the patient version of the K-MAPPIN’SDM revealed that the item-specific mean scores ranged from 2.55 to 3.55, with standard deviations between 0.68 and 1.12. The three items with the highest mean scores were Item 10: “I understood the information that doctor gave me,” Item 9: “The way the doctor exchanged information with me during the consultation suited both parties and contributed toward a mutual understanding,” and Item 11: “The doctor understood my viewpoint.” Conversely, the three items with the lowest mean scores were Item 2: “Which amongst the medical options best fits the medical problem is upon me to consider,” Item 4: “I now know the pros and cons of the different decision options,” and Item 5: “Based on the information I developed realistic expectations on probabilities benefit and harms of each option” (Fig 1).

### 3.3. Factor analysis of K-MAPPIN’SDM

The EFA of the healthcare provider version of the K-MAPPIN’SDM revealed that a KMO measure was 0.94, exceeding the minimum threshold of 0.6. Bartlett’s test of sphericity yielded statistically significant results (χ^²^ = 2460.97, *p* < 0.001). Based on the eigenvalue criterion and scree plot inspection, a dominant one-component pattern was observed. Principal component analysis confirmed that all items had factor loadings above 0.4, supporting a one-factor structure comprising all 11 items. This model accounted for 64.09% of the variance (Table 3). The CFA of the healthcare provider version of the K-MAPPIN’SDM demonstrated the following model fit indices: CFI = 0.92, TLI = 0.91, SRMR = 0.04, and RMSEA = 0.12 with a 90% confidence interval of [0.106, 0.137].Although the CFI, TLI, and SRMR met commonly recommended fit criteria, the RMSEA exceeded the recommended threshold. These findings suggest mixed evidence of model fit, and the structural validity of the healthcare provider version should therefore be interpreted with caution.

**Table 3:** Exploratory factor analysis results for the healthcare providers and patients versions of K-MAPPIN’SDM.

Items of healthcare providers version	Factors	Items of patients version	Factors
	1		1
1. The medical problem that requires a decision-making process is clear to the patient.	0.72	1. The medical problem that requires a decision-making process is clear to me.	0.56
2. Which amongst the medical options best fits the medical problem is upon the patient to consider.	0.58	2. Which amongst the medical options best fits the medical problem is upon me to consider.	0.45
3. The discussion of the options was structured in a way that is easy to understand and easy to remember. (e.g., before providing detailed information, the options were listed).	0.68	3. The discussion of the options was structured in a way that is easy to understand and easy to remember. (e.g., before providing detailed information, the options were listed).	0.71
4. The patient now knows the pros and cons of the different decision options (if applicable, also the pros and cons of the option to do without an examination and treatment).	0.82	4. I now know the pros and cons of the different decision options (if applicable, also the pros and cons of the option to do without an examination and treatment).	0.69
5. Based on the information the patient developed realistic expectations on probabilities of benefit and harms of each option.	0.85	5. Based on the information I developed realistic expectations on probabilities benefit and harms of each option.	0.71
6. The patient’s personal expectations and fears went into the decision.	0.81	6. My personal expectations and fears went into the decision.	0.46
7. At the end of the consultation it was clear to the patient why this decision was taken rather than another (If appropriate, the decision could be “to defer”).	0.86	7. At the end of the consultation it was clear to me why this decision was taken rather than another (If appropriate, the decision could be “to defer”).	0.77
8. It is now clear to the patient how his problem will in future be dealt with (e.g., who has to inform whom; when the two of us will review the decision or the deferment).	0.86	8. It is now clear to me how this problem will in future be dealt with (e.g., who has to inform whom; when the two of us will review the decision or the deferment).	0.75
9. The way I exchanged information with the patient during the consultation suited both parties and contributed toward a mutual understanding.	0.88	9. The way the health care provider exchanged information with me during the consultation suited both parties and contributed toward a mutual understanding.	0.78
10. The patient understood the information that I gave him.	0.85	10. I understood the information that health care provider gave me.	0.80
11. I understood the patient’s viewpoint.	0.84	11. The health care provider understood my viewpoint.	0.79
**Cumulative variance explained (%)**	64.09		47.82

The EFA of the patient version of the K-MAPPIN’SDM indicated that the KMO measure was 0.89, confirming the suitability of the sample for factor analysis. Bartlett’s test of sphericity confirmed statistical significance (χ^²^ = 1569.62, *p* < 0.001). Based on the eigenvalue criterion and scree plot inspection, a possible two-component pattern was observed. In the two-component solution, Items 3 and 5 exhibited cross-loadings exceeding 0.4. However, because MAPPIN’SDM was designed to evaluate each stage of SDM based on the six-step model proposed by Clayman et al. [5], item deletion was not considered appropriate according to the conceptual structure of the original instrument. Therefore, the predefined one-component structure was retained and examined. The EFA results for the one-factor model revealed that all items had factor loadings above 0.4, leading to the retention of all 11 items and confirming a one-factor structure (Table 3). This model accounted for 47.82% of the variance. The CFA of the patient version of the K-MAPPIN’SDM yielded model fit indices of CFI = 0.81, TLI = 0.76, SRMR = 0.09, and RMSEA = 0.15 with a 90% confidence interval of [0.137, 0.167]. These findings indicate that the model did not meet the commonly recommended fit criteria. Therefore, the structural validity of the patient version should be interpreted with caution.

### 3.4. Reliability and validity of K-MAPPIN’SDM

The Cronbach’s alpha coefficient for the healthcare provider version of K-MAPPIN’SDM was 0.94, and the Guttman split-half coefficient was 0.90. For the patient version, the Cronbach’s alpha coefficient was 0.87, and the Guttman split-half coefficient was 0.73.

The healthcare provider version of the K-MAPPIN’SDM presented a significant positive correlation with the SDM-Q-Doc (r = 0.88, *p* < 0.001). The patient version exhibited significant positive correlations with the SDM-Q-9 (r = 0.58, *p* < 0.001) and the CAT (r = 0.47, *p* < 0.001). These findings provide evidence for the criterion validity of both patient and healthcare provider versions of the K-MAPPIN’SDM.

## 4. Discussion

This methodological study aimed to translate and validate the MAPPIN’SDM —a tool based on a multifocal model that includes third-party observation for objective and comprehensive assessment—to better reflect the Korean clinical environment. The MAPPIN’SDM consists of three instruments designed for patients, healthcare providers, and observers, which can be used individually or in combination. In this study, we translated and adapted the instruments for patients and healthcare providers into Korean and validated their psychometric properties in Korean medical settings.

The key strength of MAPPIN’SDM lies in its ability to allow patients, providers, and observers to comprehensively evaluate the quality and outcomes of SDM. The scale for both patient and healthcare provider contains 11 items—eight core items and three optional indicators—that assess the entire SDM process from beginning to end. Each indicator is presented identically across both instruments, capturing parallel perspectives from patients and providers. Unlike conventional SDM tools that focus narrowly on treatment options and preferences, MAPPIN’SDM takes a more holistic approach [18,30], assessing the entire process, including problem definition, communication, understanding, options, and preferences in SDM.

An analysis of the mean scores for each item on the K-MAPPIN’SDM patient and healthcare provider scales showed that healthcare providers scored highest on Item 11, which measures understanding of the patient’s perspective. This suggests that healthcare providers engaged in SDM perceive themselves that they effectively empathize with their patients. On the patient instrument, Item 10 received the highest score, which assesses the patient’s understanding of the information provided. This indicates that patients felt they received and comprehended information well during SDM interactions.

However, notable differences emerged between patient and healthcare provider responses. The most significant gap was observed in the item: “Which amongst the medical options best fits the medical problem is upon me (patient) to consider (key message).” While healthcare providers acknowledge that it is up to the patient to decide which option is the most appropriate among the many approaches to solving a patient-specific problem, patients appear less confident or aware of their responsibility in decision-making. Patients’ responses to the CAT align with the findings. 46.5% of patients demonstrated a clear understanding of SDM, while 53.5% had only partial understanding. Specifically, 30.2% of the patients agreed with the SDM process but still wanted the healthcare provider to take the lead in making decisions, and 6.3% wanted the healthcare provider to make all decisions. This highlights the necessity to communicate the importance of patient involvement in decision-making more clearly and encourage patients to take ownership of their choices. As SDM is still emerging in Korea, it is essential to emphasize that no single medical option is automatically superior and that patients must be empowered to actively participate in making the best choice for themselves.

In a study by Flierler et al. [31], patients scored higher than healthcare providers on all items of the MAPPIN’SDM, but this study revealed mixed results. Patients reported higher scores than providers only on items related to communication and understanding. Providers scored higher on the remaining items. Two items were identified as weak points for both patients and healthcare providers, both related to “options”. One addressed the content of the options: “I (the patient) now know the pros and cons of the different decision options (including, if applicable, the pros and cons of choosing not to undergo an examination or treatment).” Another key question was related to the quality of information provided about the options: “Based on the information, I (the patient) developed realistic expectations regarding the probabilities of benefits and harms for each option.” To address these gaps, healthcare providers should present all available options—including “do nothing”—with a clear, balanced explanation of their potential benefits and risks. Furthermore, patients should be provided with evidence-based, clearly sourced data and comparative information to support realistic expectations and informed decision-making.

The correlations between the patient version of the K-MAPPIN’SDM and the SDM-Q-9 and CAT were moderate. This suggests that the patient version of the K-MAPPIN’SDM captures aspects of SDM that are related to, but not identical with, patient-perceived SDM and general communication quality. Therefore, the findings support criterion-related validity to some extent, but they also indicate that the instrument should not be considered interchangeable with these measures. Further testing in larger and more diverse samples, along with possible refinement of items related to options, probabilities, and patient responsibility in decision-making, may strengthen the instrument before wider use.

Regarding the factor structure of the K-MAPPIN’SDM, CFA yielded limited evidence of model fit across versions. For the healthcare provider version, CFI, TLI, and SRMR met commonly recommended criteria, whereas RMSEA and its 90% confidence interval exceeded the recommended threshold, suggesting mixed model fit. In contrast, for the patient version, CFI, TLI, SRMR, and RMSEA did not meet commonly recommended criteria, indicating inadequate model fit. This discrepancy may reflect the sensitivity of CFA fit indices to model simplicity, sample characteristics, and the process-oriented nature of the MAPPIN’SDM, in which a limited number of theoretically predefined indicators are used to capture distinct elements of the SDM process. Accordingly, the CFA results, particularly those for the patient version, should be interpreted with caution.

Given the formative and process-oriented conceptualization of the MAPPIN’SDM, CFA findings are best interpreted as supplementary rather than definitive evidence of construct validity. The primary purpose of the factor-analytic procedures in this study was not to refine or reduce items, but to examine the structural coherence of theoretically predefined SDM process indicators. Future studies should seek to replicate these findings in independent samples and further explore alternative analytic approaches that may better capture the complexity of SDM processes.

Given that awareness and understanding of SDM remain limited in Korea, the results of K-MAPPIN’SDM suggest that, specifically in the case of the patient scale, a high level of literacy is required to understand each step of SDM [9]; hence, simplification of language and guided facilitation by trained healthcare providers are recommended. Coaching by healthcare providers may enhance public understanding and acceptance of SDM, fostering greater patient participation.

This study has several limitations. First, although the study followed relevant procedures for translation, cultural adaptation, and psychometric evaluation, the COSMIN checklist was not prospectively applied as a formal methodological or reporting framework. Future studies should incorporate COSMIN-based guidance to enhance methodological transparency and comparability. Second, no formal a priori power calculation was conducted based on expected factor loadings, correlations, or model parameters. Therefore, the precision and stability of some psychometric estimates, particularly model fit indices and criterion-related validity correlations, should be interpreted cautiously. Third, uncertainty estimates such as bootstrapped confidence intervals were not reported for all key psychometric parameters. Therefore, the stability of reliability estimates, factor loadings, and criterion-related validity correlations could not be fully evaluated. Fourth, exploratory and confirmatory analyses were conducted using the same dataset, which does not constitute strict cross-validation and may increase the risk of overfitting. Therefore, the CFA findings should be interpreted as preliminary evidence rather than definitive confirmation of the factor structure. Fifth, although the healthcare provider dataset included 288 SDM encounters, these observations were nested within a small number of physicians. This clustering was not accounted for in the modelling and may have affected the precision of estimates and limited the generalizability of the findings. Because the number of physician clusters was small, more complex multilevel modelling was not performed in this validation study. Future studies should include a larger number of physicians and clinical sites and apply analytic approaches that account for clustering.

In addition, the study was conducted in three specialties (i.e., endocrinology, nephrology, and orthopedics) at a single university hospital in Korea, with physician participation based on voluntary consent during the data collection period, which limits the generalizability of the findings. Future studies should include more diverse and representative samples. Although age-related differences in SDM were not examined in the present validation study, cultural factors such as family involvement and collectivist tendencies, as noted in prior Korean research, may influence how SDM is perceived and enacted across age groups. Future studies should explicitly examine these patterns using designs tailored to cultural and demographic comparisons. Finally, the instruments validated in this study are self-assessment tools that evaluate the SDM process separately for patients and healthcare providers. Future research should incorporate the observer scale to allow for a comprehensive evaluation of SDM quality and outcomes, and we are currently in the process of developing it. Moving forward, it is essential to develop tools that both support and evaluate SDM, establish standardized implementation procedures, and incorporate observer evaluation into clinical practice. Equally important is the development of structured training programs to ensure inter-rater reliability among observers, along with policy-level support for widespread SDM implementation.

### 4.1. Practice implications

The K-MAPPIN’SDM assessment tool has several significant implications for clinical practice. First, it provides a comprehensive framework for evaluating SDM implementation through multiple perspectives. This multi-perspective approach enables a more complete evaluation of SDM’s impact on clinical outcomes. Second, the K-MAPPIN’SDM can serve as a valuable research instrument for identifying the current state of SDM implementation and analyzing differences in perspectives between patients and healthcare providers. It will also function as an essential evaluation tool for the Korean SDM model currently under development. Third, since the MAPPIN’SDM is structured on the established 6-step SDM model, it offers an effective framework for training healthcare providers in SDM practices. Furthermore, as healthcare systems work toward institutionalizing SDM, the K-MAPPIN’SDM will help generate the scientific evidence needed to demonstrate SDM’s effects on healthcare quality, patient satisfaction, and health outcomes.

## 5. Conclusion

The K-MAPPIN’SDM developed in this study provides a comprehensive framework for assessing SDM by integrating patients’ values with healthcare providers’ clinical expertise. The instrument supports the evaluation of SDM implementation in routine clinical practice, serves as a research and training tool, and has the potential to contribute to improvements in healthcare quality, patient autonomy, and clinical outcomes in the Korean healthcare context.

Beyond its practical utility, this study contributes to the existing body of knowledge on SDM measurement in several important ways. First, it extends the applicability of the MAPPIN’SDM framework to a non-Western healthcare context through rigorous and culturally sensitive translation and validation, addressing the need for culturally appropriate SDM assessment tools. Second, by validating both patient and healthcare provider versions, this study highlights the multidimensional and process-oriented nature of SDM, emphasizing that SDM is best understood through complementary perspectives rather than a single rater. Importantly, the meaning of divergent appraisals of involvement across multiple SDM indicators between patients and healthcare providers is not yet fully understood, underscoring the need for further evaluation, particularly through the inclusion of an observer perspective. Third, this study highlights methodological challenges in evaluating SDM instruments, as process-oriented tools such as the MAPPIN’SDM are not always well represented by traditional factor-analytic models that assume item homogeneity. Together, these contributions advance the methodological and conceptual foundations for SDM research and provide a basis for future studies examining SDM implementation, training, and outcomes in diverse cultural and clinical settings.

## Supporting information

S1 Checklist Inclusivity in global research. (PDF)
