# Supplementary material for: Reliability and validity of the Korean version of MAPPIN’SDM for patients and healthcare providers
Source: PLoS One. 2026 Sep 3;21(9):e0347018. doi: 10.1371/journal.pone.0347018 (PMC13541307; doi:10.1371/journal.pone.0347018)
Supplement: Supplementary file 3 [file pone.0347018.s003.docx]

Thank you very much for your valuable feedback. The sections that have been revised in the manuscript are highlighted in red for emphasis.

1. **Reviewer 1**

R1-1.  The introduction section must end with the research question/s or hypothesis

- **RESPONSE:** Thank you for this comment. We have revised the Introduction to ensure that the study aim is clearly stated at the end of the section.
  - *As an initial phase of this research, this study focused on the translation and validation of the patient and healthcare provider versions of the instrument. By evaluating the psychometric properties of these two instruments, this study seeks to establish a reliable and valid tool for measuring SDM in Korea, ultimately contributing to the development of a comprehensive SDM model and assessment framework tailored to the Korean healthcare environment. This study aimed to develop and validate the Korean version of the MAPPIN’SDM (K-MAPPIN’SDM) to assess SDM in Korean clinical settings. [Introduction]*

R1-2.  The discussion section must have a comparison table comparing this work with other related works.

- **RESPONSE:** Thank you for this comment. We agree that situating the present findings within the broader SDM literature is important. However, the current study was designed as a translation and validation study of the Korean version of the MAPPIN’SDM, with a primary focus on its psychometric properties rather than on comparative evaluation across instruments.

Accordingly, we did not include a comparison table in the Discussion, as such a table would require predefined and potentially arbitrary comparison criteria and could detract from the main objective of the validation study. Instead, we have contextualized our findings through narrative comparison with relevant prior work in the Discussion, focusing on conceptual alignment and methodological considerations rather than tabulated contrasts. We believe this approach is more appropriate for the scope and aims of the present study.

R1-3. The conclusion section is too short and needs a paragraph on contributions to the body of knowledge.

- **RESPONSE:** We have expanded the Conclusion section to explicitly articulate the study’s contributions to the body of knowledge, including its conceptual, methodological, and contextual significance for SDM measurement.
- *The K-MAPPIN’SDM developed in this study provides a comprehensive framework for assessing SDM by integrating patients’ values with healthcare providers’ clinical expertise. The instrument supports the evaluation of SDM implementation in routine clinical practice, serves as a research and training tool, and has the potential to contribute to improvements in healthcare quality, patient autonomy, and clinical outcomes in the Korean healthcare context.*

*Beyond its practical utility, this study contributes to the existing body of knowledge on SDM measurement in several important ways. First, it extends the applicability of the MAPPIN’SDM framework to a non-Western healthcare context through rigorous and culturally sensitive translation and validation, addressing the need for culturally appropriate SDM assessment tools. Second, by validating both patient and healthcare provider versions, this study highlights the multidimensional and process-oriented nature of SDM, emphasizing that SDM is best understood through complementary perspectives rather than a single rater. Importantly, the meaning of divergent appraisals of involvement across multiple SDM indicators between patients and healthcare providers is not yet fully understood, underscoring the need for further evaluation, particularly through the inclusion of an observer perspective. Third, this study highlights methodological challenges in evaluating SDM instruments, as process-oriented tools such as the MAPPIN’SDM are not always well represented by traditional factor-analytic models that assume item homogeneity. Together, these contributions advance the methodological and conceptual foundations for SDM research and provide a basis for future studies examining SDM implementation, training, and outcomes in diverse cultural and clinical settings. [Conclusion]*

R1-4. The associated statistics must involve enough rigor in terms of graphs and charts which is missing.

- **RESPONSE:** Thank you for this thoughtful comment. We agree that clear graphical and tabular presentation is important for **effectively conveying statistical findings**. In the current manuscript, we have sought to address this by including detailed tables and figures that support the statistical analyses and enhance transparency and interpretability. Specifically, the results of the exploratory factor analyses for both the healthcare provider and patient versions are presented in comprehensive tables that include factor loadings and related statistics. In addition, Figure 1 offers a graphical comparison of average MAPPIN’SDM item scores between healthcare providers and patients, allowing readers to examine item-level patterns across respondent groups. **Given the validation-focused aims of the study, we believe that the existing combination of tables and figures provides adequate graphical support for the analyses conducted.**

R1-5. A conceptual diagram describing the problem domain at the end of the introduction section can help.

- **RESPONSE:** Thank you for this suggestion. We agree that conceptual diagrams can be useful in studies aiming to introduce or develop new theoretical frameworks. However, the present study was designed as a translation and validation study of an existing instrument rather than as a conceptual or theory-building investigation.

The conceptual domain of shared decision-making and the structure of the MAPPIN’SDM are already well established in prior literature and in the original instrument. Accordingly, we focused the Introduction on clearly describing the conceptual background and measurement rationale in narrative form, which we believe is more appropriate for the scope and aims of this validation-focused study. For this reason, we did not include an additional conceptual diagram.

2. **Reviewer 2**

R2-1.  [Introduction] The introduction was well-written the need of study. The authors state that both the Korean-translated SDM-Q-9 and the scale developed by Jo are the most widely used tools in South Korea. However, although the manuscript summarizes Jo’s study, it does not discuss the limitations of Park et al.’s work. Please add a description of those limitations.

- **RESPONSE:** Thank you for this helpful comment. We agree that the limitations of existing SDM instruments should be clearly articulated. In response, we have revised the Introduction to address limitations of the Korean versions of the SDM-Q-9. Specifically, we note that no validation studies have been published on the Korean version of the SDM-Q-9, and that, unlike the MAPPIN’SDM, it does not assess key process-level elements such as the structured presentation of treatment options, the quality and balance of information, and communication behaviors involved in integrating patient preferences. This revision clarifies the rationale for the present study and the need for a process-oriented SDM assessment tool.
- *The SDM-Q-9 also has notable limitations. Unlike the MAPPIN’SDM, it does not systematically assess each steps of the SDM process, such as defining the problem, explaining the benefits and harms of each option, exploring patient preference and needs, and indicating the decision. Furthermore, no validation studies have been published on the Korean version of the SDM-Q-9 yet. [Introduction]*

R2-2. [Methods] Please explain the rationale or reasons for restricting the study to three clinical departments.

- **RESPONSE:** Thank you for raising this point. Recruitment was not restricted to specific departments in advance. During the recruitment period, physicians from the departments of endocrinology, orthopedics, and nephrology agreed to participate in the study, and patients receiving care in these departments were subsequently recruited. We have clarified the recruitment process in the Methods section and acknowledge the resulting limitation in generalizability in the Discussion.
  - *During the recruitment period, physicians from the departments of endocrinology, orthopedics, and nephrology agreed to participate in the study, and patients receiving care in these departments were subsequently recruited. [Methods]*
  - *Participation was voluntary, and only physicians from departments that agreed to participate, along with patients receiving care in those departments, were included in the study. [Methods]*
  - *First, the study was conducted in three specialties (i.e., endocrinology, nephrology, and orthopedics) at a single university hospital in Korea, with physician participation based on voluntary consent during the data collection period, which limits the generalizability of the findings. [Discussion]*

R2-3.  [Methods] The manuscript applies EFA and CFA to the same sample, which runs counter to the cross-validation principle and increases the risk of overfitting. This should be addressed—ideally by re-estimating the CFA in an independent sample (e.g., split-sample, holdout, or separate cohort), or at minimum by clearly acknowledging it as a limitation. In addition, the healthcare-professional subsample appears very small (n=6), which undermines the stability of factor loadings and limits generalizability; please report the exact N, justify sample-size adequacy (e.g., subject-to-item ratio), and discuss implications. Finally, in the patient-version CFA, at least one fit index (e.g., RMSEA) did not meet commonly accepted thresholds (e.g., < 0.08). Although the value is reported in the Results, the Discussion does not interpret its statistical/substantive implications nor list it as a limitation. Please provide a balanced interpretation of model fit (RMSEA with CI, CFI/TLI, SRMR), justify the thresholds used, and either improve the model or explicitly acknowledge the limitation.

- **RESPONSE:** We thank the reviewer for the careful methodological comments and appreciate the opportunity to clarify several points.

**Use of EFA and CFA in the same sample**

- We acknowledge that using the same dataset for exploratory and confirmatory analyses does not constitute strict cross-validation and may increase the risk of overfitting in reflective latent variable modeling. In the present study, however, the factor structure of MAPPIN’SDM was not derived inductively from the data but was theoretically predefined based on the original instrument. The exploratory analysis was used to examine structural coherence rather than to discover latent dimensions, and the CFA was conducted as a supplementary evaluation of model fit. Nevertheless, we agree that replication in an independent sample would strengthen the evidence and have explicitly acknowledged this as a limitation.

**Clarification of the healthcare provider sample size**

- We would like to clarify that although six physicians participated, the unit of analysis for the healthcare provider version was the SDM encounter, not the individual physician. Each physician completed the MAPPIN’SDM healthcare provider questionnaire repeatedly across outpatient consultations, resulting in 288 healthcare provider-rated SDM encounters. We have revised the Methods section to clarify this analytic structure. We also acknowledge that these encounter-level observations are nested within physicians, and we have added this consideration to the Discussion as a limitation.

**Interpretation of CFA model fit**

- We agree that the RMSEA exceeded commonly cited thresholds. In the revised Discussion, we now provide a balanced interpretation of model fit by reporting RMSEA with its confidence interval alongside CFI, TLI, and SRMR. While the incremental and absolute fit indices suggested acceptable fit, the elevated RMSEA may reflect model simplicity and sensitivity to sample characteristics. This issue is now explicitly discussed as a limitation rather than overlooked.

Overall, the manuscript has been revised to improve transparency regarding analytic decisions, clarify the effective sample size for provider analyses, and provide a balanced interpretation of CFA results.

- *Exploratory and confirmatory analyses were conducted using the same dataset. The exploratory analysis was intended to examine the structural coherence of the predefined indicators rather than to inductively derive latent dimensions, and the confirmatory analysis was performed as a supplementary evaluation of model fit based on the theoretically established structure of the original instrument. [Methods]*
- *For the healthcare provider version, the unit of analysis was the SDM encounter rather than the individual physician. Although six physicians participated, each physician completed the healthcare provider version of the MAPPIN’SDM across multiple outpatient consultations, resulting in 288 healthcare provider–rated SDM encounters. [Methods]*
- *The CFA of the healthcare provider version of the K-MAPPIN’SDM demonstrated the following model fit indices: CFI = 0.81, TLI = 0.76, SRMR = 0.09, and RMSEA = 0.15 with a 95% confidence interval of [0.056, 0.090]. [Results]*
- *The CFA of the patient version of the K-MAPPIN’SDM yielded model fit indices of CFI = 0.92, TLI = 0.91, SRMR = 0.04, and RMSEA = 0.12 with a 95% confidence interval of [0.114, 0.145]. [Results]*
- *Regarding the factor structure of the K-MAPPIN’SDM, CFA yielded mixed model fit across versions. Although CFI, TLI, and SRMR suggested acceptable model fit, the RMSEA and its confidence interval exceeded commonly recommended thresholds. This discrepancy may reflect the sensitivity of RMSEA to model simplicity and sample characteristics, particularly in the context of a process-oriented instrument with a limited number of indicators. Accordingly, the CFA results should be interpreted with caution.*
- *Given the formative and process-oriented conceptualization of the MAPPIN’SDM, CFA findings are best interpreted as supplementary rather than definitive evidence of construct validity. The primary purpose of the factor-analytic procedures in this study was not to refine or reduce items, but to examine the structural coherence of theoretically predefined SDM process indicators. Future studies should seek to replicate these findings in independent samples and further explore alternative analytic approaches that may better capture the complexity of shared decision-making processes. [Discussion]*
- *Second, exploratory and confirmatory analyses were conducted using the same dataset, which does not constitute strict cross-validation and may increase the risk of overfitting. Although the factor structure was theoretically predefined, replication in an independent sample is warranted. Third, although the healthcare provider dataset included 288 SDM encounters, these observations were nested within a small number of physicians. This clustering may limit generalizability and should be considered when interpreting the results. [Discussion]*

R2-4.  [Methods] In the clinician sample (n=6), one participant reported prior SDM training. This could confound clinician-reported SDM scores. Please report outcomes by training status and conduct a sensitivity analysis (e.g., excluding the trained clinician). At minimum, compare the trained clinician’s scores with the other five and discuss implications for generalizability?

- **RESPONSE:** We appreciate the reviewer’s thoughtful comment regarding prior SDM training. We agree that, in principle, clinician training may influence SDM-related behaviors. However, the present study was designed as a validation study rather than an evaluation of training effects. The primary objective was to examine the structural properties of the Korean version of the MAPPIN’SDM, not to compare SDM performance across clinician subgroups.

We further note that the reported training was based on a single self-reported item and did not specify whether the training was a standardized SDM program or more general communication-related education, which is common in the current Korean clinical context. Given that only one clinician reported prior training, meaningful subgroup comparison or sensitivity analysis would not be statistically informative.

Moreover, because the unit of analysis for the healthcare provider version was the SDM encounter rather than the individual clinician, conducting encounter-level comparisons by training status could conflate individual clinician response styles with training effects, potentially yielding misleading or spurious findings. For this reason, we considered such analyses inappropriate for the aims of the present validation study.

R2-5.  [Methods] Jo’s culturally adapted SDM instrument suggests that Korean decision making often incorporates family interests and collectivist tendencies. The age composition of participants could shape these responses. Please (1) provide detailed age characteristics (range, mean±SD, and proportions by age bands), (2) assess age-related differences in SDM (e.g., <40, 40–64, ≥65 or similar), either via stratified analyses or by including age as a covariate, and (3) discuss whether any observed pattern is consistent with the family-centered tendencies described by Jo.

- **RESPONSE:** We appreciate the reviewer’s insightful comment regarding the potential influence of age and collectivist decision-making tendencies in the Korean context, as discussed by Jo. We agree that age may shape how shared decision-making is experienced and enacted in clinical encounters.

However, the present study was designed as a validation study of the Korean version of the MAPPIN’SDM, with the primary objective of examining the structural properties of the instrument rather than evaluating age-related differences in SDM performance. Accordingly, age was not treated as an explanatory variable, and stratified or covariate-based analyses were beyond the intended scope of this study.

We have provided detailed descriptive age characteristics of the sample to enhance transparency. In the Discussion, we now acknowledge that age-related patterns in SDM, including potential family-centered decision-making tendencies, represent an important direction for future research. Such questions would be better addressed in studies specifically designed to examine cultural and demographic influences on SDM, incorporating variables that directly capture family involvement and decision-making roles.

- *The age distribution of patients was as follows: 18–29 years (n = 28), 30–39 years (n = 33), 40–49 years (n = 61), 50–59 years (n = 61), 60–69 years (n = 76), and 70 years or older (n = 29). [Results]*
- In addition, although age-related differences in SDM were not examined in the present validation study, cultural factors such as family involvement and collectivist tendencies, as noted in prior Korean research, may influence how SDM is perceived and enacted across age groups. Future studies should explicitly examine these patterns using designs tailored to cultural and demographic comparisons. [Discussion]

R2-6.  [Methods] The EFA was implemented via PCA, which is a data-reduction technique rather than a common factor model. Unless the sole aim is reduction, please re-estimate the EFA using a common factor extraction on an appropriate correlation matrix for ordinal items (e.g., polychoric), with oblique rotation. Because the one-factor solution was not supported and two factors emerged, determine dimensionality using parallel analysis in addition to the scree plot, and report retention criteria, communalities, and cross-loadings.

- **RESPONSE:** Thank you for the reviewer’s constructive comments on the exploratory analysis. We acknowledge that principal component analysis (PCA) is commonly described as a data-reduction method and differs from common-factor extraction used to model latent reflective constructs.

In this study, however, the MAPPIN’SDM is conceptualized as a formative set of theoretically essential and non-interchangeable indicators of the SDM process. Each indicator is represented by a single item, and all indicators are retained by design to preserve content validity and the conceptual integrity of the instrument. Accordingly, our analytic aim was not latent factor identification or item reduction, but to examine the structural coherence among these predefined process indicators. For this purpose, PCA was considered appropriate, and we therefore did not adopt common-factor extraction methods (e.g., polychoric-based EFA) that would impose reflective measurement assumptions.

To facilitate interpretation of the component structure and to obtain a clearer separation among components, we applied an orthogonal Varimax rotation. Salient loadings were interpreted using a threshold of ≥ 0.40, and we have added communalities and cross-loadings, along with the component retention rationale, to the revised manuscript for transparency.

Finally, we respectfully note that procedures aimed at determining the optimal number of latent dimensions (e.g., scree plot inspection and parallel analysis) are primarily intended for reflective latent-variable modeling and dimensional reduction. Because the number of indicators in MAPPIN’SDM was fixed a priori and not subject to empirical reduction, we did not use these procedures to guide retention. Any multi-component pattern was interpreted as clustering among distinct SDM process elements rather than evidence of separable latent constructs. We have revised the Methods to clarify these conceptual and methodological decisions.

- *EFA was conducted to examine the structural coherence of the predefined indicators. The Kaiser–Meyer–Olkin (KMO) measure and Bartlett’s test of sphericity were used solely to assess the suitability of the data for exploratory analysis. To examine the structural coherence among the predefined indicators of the shared decision-making process, principal component analysis (PCA) was conducted. The MAPPIN’SDM is conceptualized as a formative measurement instrument in which each item represents a distinct and theoretically essential SDM process indicator. Because the indicators are non-interchangeable and retained by design, no item was considered for deletion and dimensional reduction procedures were not applied.*

*For clearer interpretation and separation of the component structure, an orthogonal Varimax rotation was employed. Loadings of ≥ 0.40 were considered salient for interpretative purposes. Communalities and cross-loadings were examined to evaluate the contribution of each indicator to the component structure.*

*Because the number of indicators was fixed a priori and not subject to empirical reduction, factor-retention procedures intended to select the optimal number of latent dimensions (e.g., scree plot inspection or parallel analysis) were not used to guide retention. Any emergence of multiple components was interpreted as clustering among distinct SDM process elements rather than evidence of separable latent constructs. [Methods]*

R2-7.  [Methods] The expression “somewhat poor model fit” is not sufficiently informative for scholarly reporting. Please specify the a priori decision rules for model adequacy, present the observed indices (RMSEA with 90% CI, CFI, TLI, SRMR), justify the cutoffs, and discuss the plausible reasons for the suboptimal fit.

- **RESPONSE:** Thank you for this helpful comment. We agree that the phrase “somewhat poor model fit” was imprecise and insufficient for scholarly reporting. In the revised manuscript, we have removed this qualitative description and replaced it with explicit a priori decision rules for model adequacy and detailed reporting of observed fit indices.

In addition to the revisions described above, we revised the Abstract and the Design section to improve clarity and logical flow.
