# Supplementary material for: Reliability and validity of the Korean version of MAPPIN’SDM for patients and healthcare providers
Source: PLoS One. 2026 Sep 3;21(9):e0347018. doi: 10.1371/journal.pone.0347018 (PMC13541307; doi:10.1371/journal.pone.0347018)
Supplement: Supplementary file 4 [file pone.0347018.s004.docx]

Thank you very much for your valuable feedback. The sections that have been revised in the manuscript are highlighted in red for emphasis.

1. **Reviewer 3**

R1.  There is a research checklist (COSMIN) the authors could be using, although they are using some relevant guidelines. They need to discuss this as a potential limitation.

- **RESPONSE:** Thank you for this important comment. We agree that COSMIN provides a useful framework for improving methodological rigor and reporting transparency in studies evaluating measurement properties. Although our study followed relevant procedures for translation, cultural adaptation, and psychometric evaluation, we did not prospectively apply the COSMIN checklist as a formal methodological or reporting framework. We have added this issue as a limitation in the Discussion.
  - *Although the study followed relevant procedures for translation, cultural adaptation, and psychometric evaluation, the COSMIN checklist was not prospectively applied as a formal methodological or reporting framework. Future studies should incorporate COSMIN-based guidance to enhance methodological transparency and comparability. [Discussion]*

R2.  There is no formal power calculation reported. Instead, the sample size is justified using general rules of thumb for factor analysis. Although this is common, a more robust approach would base this on the ability to reliably detect expected correlations or factor loadings. This needs to be discussed as a limitation.

- **RESPONSE:** We agree with the reviewer. No formal a priori power calculation was conducted based on expected factor loadings, correlations, or model parameters. We have revised the Discussion to acknowledge this as a limitation and to clarify that the precision and stability of some psychometric estimates should be interpreted cautiously.
  - *No formal a priori power calculation was conducted based on expected factor loadings, correlations, or model parameters. Therefore, the precision and stability of some psychometric estimates, particularly model fit indices and criterion-related validity correlations, should be interpreted cautiously. [Discussion]*

R3. Linked to the previous point, it would strengthen the paper to report uncertainty around all key results, for example using bootstrapping. This could partially compensate for the lack of a formal power calculation by showing how stable the results are.

- **RESPONSE:** Thank you for this helpful suggestion. We agree that reporting uncertainty estimates, such as bootstrapped confidence intervals, would strengthen the interpretation of the psychometric findings. In the present study, bootstrapped confidence intervals were not reported for all key psychometric parameters. We have therefore acknowledged this as a limitation and tempered the interpretation of the psychometric findings accordingly.
- *Uncertainty estimates such as bootstrapped confidence intervals were not reported for all key psychometric parameters. Therefore, the stability of reliability estimates, factor loadings, and criterion-related validity correlations could not be fully evaluated. [Discussion]*

R4. The manuscript does not report the extent of missing data or how missing values were handled, which limits assessment of potential bias. Please provide the level, pattern, and handling of missing data, and justify whether imputation methods were necessary.

- **RESPONSE:** Thank you for pointing this out. We have clarified the extent and handling of missing data in the Methods section. There were no missing values in the variables included in the psychometric analyses; therefore, no imputation or case deletion due to missingness was required.
- *Before the main analyses, the dataset was screened for missing values. No missing values were identified in the variables included in the psychometric analyses; therefore, no case deletion due to missingness or imputation procedure was required. [Methods]*

R5. There are only 6 physicians so the data are heavily clustered. This is not really discussed as a limitation and not accounted for in modelling. Strong within correlations will introduce bias.

- **RESPONSE:** We agree with the reviewer. Although the study included 288 SDM encounters, these encounters were nested within only six physicians. We acknowledge that this clustering was not accounted for in the modelling and may have affected the precision of estimates and the generalizability of the findings. Because the number of physician clusters was small, more complex multilevel modelling was not performed in this validation study. We have expanded the limitation section accordingly.
  - *Although the healthcare provider dataset included 288 SDM encounters, these observations were nested within a small number of physicians. This clustering was not accounted for in the modelling and may have affected the precision of estimates and limited the generalizability of the findings. Because the number of physician clusters was small, more complex multilevel modelling was not performed in this validation study. Future studies should include a larger number of physicians and clinical sites and apply analytic approaches that account for clustering. [Discussion]*

R6. Some of the key validity results are only moderate, suggesting the tool is not capturing the same concepts very strongly across measures. While reliability Cronbach’s alpha is good and above the usual 0.7 threshold, the weaker correlations should be discussed more clearly in terms of what they mean for how well the tool measures shared decision-making.

- **RESPONSE:** Thank you for this comment. We agree that the moderate correlations observed for the patient version require more careful interpretation. We have revised the Results and Discussion to clarify that the K-MAPPIN’SDM patient version is related to, but not interchangeable with, patient-reported SDM and general communication measures.
  - *The correlations between the patient version of the K-MAPPIN’SDM and the SDM-Q-9 and CAT were moderate. This suggests that the patient version of the K-MAPPIN’SDM captures aspects of SDM that are related to, but not identical with, patient-perceived SDM and general communication quality. Therefore, the findings support criterion-related validity to some extent, but they also indicate that the instrument should not be considered interchangeable with these measures. [Discussion]*

R7. Overall, the instrument appears usable, but these modest results suggest it may need refinement in certain aspects. Further testing in larger and more diverse samples, and possibly improving some items, would strengthen confidence before wide use. This needs to be discussed appropriately.

- **RESPONSE:** We agree. We have revised the Discussion to state that further testing in larger and more diverse samples is needed before wider use. We also noted that possible refinement of items related to options, probabilities, and patient responsibility in decision-making may strengthen the instrument.
  - *Further testing in larger and more diverse samples, along with possible refinement of items related to options, probabilities, and patient responsibility in decision-making, may strengthen the instrument before wider use. [Discussion]*

R8. Model fit indices, e.g., RMSEA, do not meet standard thresholds but are only briefly discussed. This should be more clearly interpreted and acknowledged as a limitation.

- **RESPONSE:** Thank you for this important point. We have revised the Results and Discussion to more clearly interpret the model fit indices. Specifically, the healthcare provider version showed mixed evidence of model fit, with acceptable CFI, TLI, and SRMR but elevated RMSEA. In contrast, the patient version did not meet commonly recommended fit criteria across the major indices. We have therefore acknowledged the limited CFA model fit as a limitation and tempered the interpretation of structural validity.
  - *Regarding the factor structure of the K-MAPPIN’SDM, CFA yielded limited evidence of model fit across versions. For the healthcare provider version, CFI, TLI, and SRMR met commonly recommended criteria, whereas RMSEA and its 90% confidence interval exceeded the recommended threshold, suggesting mixed model fit. In contrast, for the patient version, CFI, TLI, SRMR, and RMSEA did not meet commonly recommended criteria, indicating inadequate model fit. This discrepancy may reflect the sensitivity of CFA fit indices to model simplicity, sample characteristics, and the process-oriented nature of the MAPPIN’SDM, in which a limited number of theoretically predefined indicators are used to capture distinct elements of the SDM process. Accordingly, the CFA results, particularly those for the patient version, should be interpreted with caution. [Discussion]*

R9. EFA and CFA are conducted on the same dataset, which increases the risk of overfitting. This should be more explicitly acknowledged as a limitation.

- **RESPONSE:** We agree with the reviewer. We have revised the limitation section to explicitly state that conducting exploratory and confirmatory analyses using the same dataset does not constitute strict cross-validation and may increase the risk of overfitting. We also clarified that the CFA findings should be interpreted as preliminary rather than definitive evidence of the factor structure.
  - *Exploratory and confirmatory analyses were conducted using the same dataset, which does not constitute strict cross-validation and may increase the risk of overfitting. Therefore, the CFA findings should be interpreted as preliminary evidence rather than definitive confirmation of the factor structure. [Discussion]*
